# A novel hydroxyfuroic acid compound as an insulin receptor activator - structure and activity relationship of a prenylindole moiety to insulin receptor activation

**DOI:** 10.1186/1423-0127-16-68

**Published:** 2009-07-30

**Authors:** Henry J Tsai, Shan-Yen Chou

**Affiliations:** 1 Pharmaceutical R&D Program, Development Center for Biotechnology, Hsi-Chih City 221, Taiwan, Republic of China; 2 Department of Biological Science and Technology, China Medical University, Taichung City 404, Taiwan, Republic of China; 3 Department of Health and Nutrition Biotechnology, Asia University, Taichung County 413, Taiwan, Republic of China; 4 Taigen Biotechnology Co, Taipei 114, Taiwan, Republic of China

## Abstract

**Background:**

Diabetes Mellitus is a chronic disease and many patients of which require frequent subcutaneous insulin injection to maintain proper blood glucose levels. Due to the inconvenience of insulin administration, an orally active insulin replacement has long been a prime target for many pharmaceutical companies. Demethylasterriquinone (DMAQ) B1, extracted from tropical fungus, *Pseudomassaria *sp., has been reported to be an orally effective agent at lowering circulating glucose levels in diabetic (*db/db*) mice; however, the cytotoxicity associated with the quinone moiety has not been addressed thus far.

**Methods:**

A series of hydroxyfuroic acid compounds were synthesized and tested for their efficacies at activating human insulin receptor. Cytotoxicity to Chinese hamster ovary cells, selectivities over insulin-like growth factor-1 (IGF-1), epidermal growth factor (EGF), and fibroblast growth factor (FGF) receptors were examined in this study.

**Result and Conclusion:**

This study reports a new non-quinone DMAQ B1 derivative, a hydroxyfuroic acid compound (D-410639), which is 128 fold less cytotoxic as DMAQ B1 and as potent as compound 2, a DMAQ B1 synthetic derivative from Merck, at activating human insulin receptor. D-410639 has little activation potential on IGF-1 receptor but is a moderate inhibitor to EGF receptor. Structure and activity relationship of the prenylindole moiety to insulin receptor activation is discussed.

## Background

Diabetes mellitus is a chronic disease characteristic of elevated blood glucose concentrations with poor glucose utilization and homeostasis [1]. About 10% of all diabetic patients are type 1 insulin dependent diabetes mellitus (IDDM) in which insulin secreting β-islets of Langerhans are damaged or destroyed by aberrant T cells [2]. Other diabetes cases (about 90%) are type 2 non-insulin dependent diabetes mellitus (NIDDM) that is proceeded by insulin resistance and frequently with metabolic syndrome [1]. For type 1 and late stage type 2 diabetic patients, a common method of alleviating hyperglycemia is by subcutaneous administration of exogenous insulin before each meal [1,2]. Due to the inconvenience of insulin administration, it has long been a primary goal of many pharmaceutical companies to develop an orally active therapeutic agent for treating hyperglycemia in diabetic patients.

Current diabetes therapies with orally active agents fall into five major classes, which are i) biguanide (metformin) that activates AMP-activated protein kinase (AMPK) [3-6]; ii) sulfonylurea as an insulin secretogue [7-9]; iii) peroxisome proliferator activated receptor (PPAR) γ-subtype activators [10-12]; iv) α-glucosidase inhibitors [13,14]; v) dipeptidyl peptidase IV (DP-4) inhibitors [15-18]. In addition to these targets, the insulin receptor activator is particularly interesting, because it may activate the insulin signal transduction pathway directly without the need of insulin, and yet it is small enough to be orally active. Extracted from tropical fungus, *Pseudomassaria *sp., demethylasterriquinone (DMAQ) B1 is one such compound [19-24] and has been shown to lower blood glucose in *db/db *mice by activating insulin receptor's tyrosine kinase directly [19,22]. The compound was later modified to phenylindolyldihydroxyquinone (compound 2, by Merck's nomenclature), with an improved efficacy (EC_50 _from 5.0 μM to 0.3 μM) [20,21]. However, DMAQ B1 and compound 2 each has a hydroxyquinone moiety that may facilitate free radical generation when in contact with high energy electrons [25]. Therefore, a new insulin receptor activator without a quinone moiety is a logical compound to develop.

**Figure 1:**
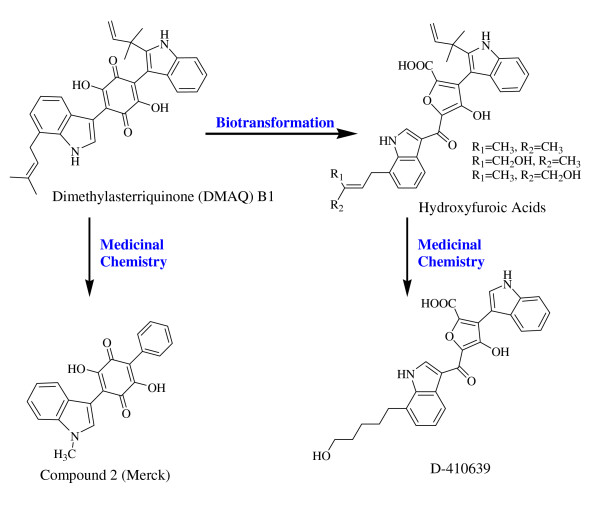
**Active receptor tyrosine kinase activators**. Demethylasterriquinone B1 is extracted from tropical fungus, *Pseudomassaria *sp., and can be transformed to hydroxyfuroic acid which still retains insulin receptor activation capability. Compound 2 and D-410639 are derived from Demethylasterriquinone B1 and hydroxyfuroic acid, respectively.

It has been reported that DMAQ B1 can be converted to bisindolylhydroxyfuroic acids by biotransformation and thus replaces its quinone with a furoic acid moiety (Fig. 1), but still retains its insulin receptor activation potential [Chen *et al. *US Patent 6596760, 2003]. While through medicinal chemistry, prenylindole and isoprenylindole moieties on DMAQ B1 can be simplified to an indolyl and a phenyl moieties resulting a phenylindolyldihydroxyquinone (compound 2, Fig. 1) with an improved efficacy [20,21]. Combining these two features together, phenylindolylfuroic acid derivatives were synthesized [26], but for unknown reasons these compounds showed no observable insulin receptor activation efficacy (data not shown). Therefore, these phenylindolylfuroic acid derivatives were not pursued further, and the bisindolylfuroic acid scaffold is retained for further derivative development because of its absence of a quinone moiety. The isoprenyl chain on the isoprenylindole is not essential for the insulin receptor activation in our setting, and was omitted in subsequent derivatives (Fig. 1). A new class of insulin receptor activators was discovered under such circumstances. We report a new hydroxyfuroic acid compound, D-410639, that possesses insulin receptor activation property as well as inhibition for epidermal growth factor receptor (EGF-R/ErbB1).

## Methods

### Materials and Chemicals

The CHO cell line overexpressing recombinant human insulin receptor was kindly provided by Dr. Richard Roth at Stanford University. Scintillation cocktail and γ-P^33 ^ATP were purchased from Perkin-Elmer (Wellesley, MA). P81 phosphocellulose paper is a product of Millipore (Billerica, MA). Ab-3 antibody is a product of Upstate (Charlottesville, VA). Recombinant human IGF-1 receptor, EGF-R (ErbB1), and FGF-R3 were purchased from R&D systems (Minneapolis, MN). DMAQ B1 and Compound 2 of Merck were obtained from Calbiochem (La Jolla, CA). Other chemicals were purchased from Sigma-Aldrich (St. Louis, MO).

### Syntheses of Hydroxyfuroic Acid Compounds

**Figure 2:**
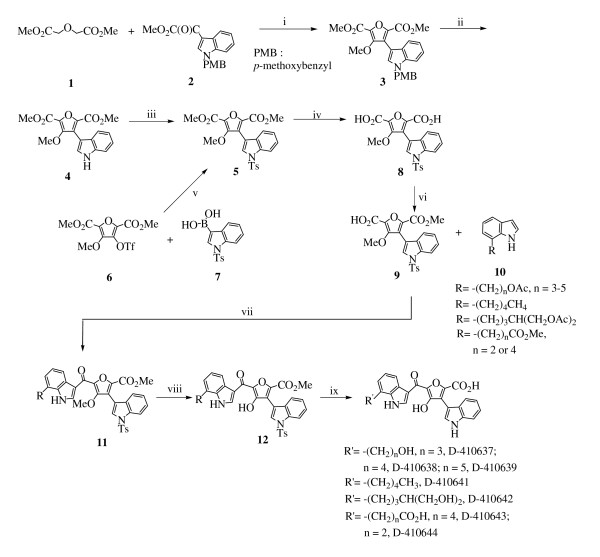
**Preparation of bisindolylhydroxyfuroic acids**. i. a) K, *t*-BuOH, benzene; b) MeI, *N,N*-dimethylformamide (DMF), 45% (two steps); ii. DDQ, CH_2_Cl_2_/H_2_O (18/1, v/v); 46%; iii. TsCl, 2-butanone, K_2_CO_3_, reflux, 86%; iv. OH^-^, MeOH, H_2_O, 95%; v. PdCl_2 _(PPh_3_)_2 _(0.1 eq), Na_2_CO_3 _(3.0 eq), DMF, 90°C, 12 h, 66%; vi. ClCO_2_Me (100 mol %), NEt_3 _(107 mol %), CH_2_Cl_2 _(6 mL/mmol **8**), DMAP (12 mol %), 60%; vii. a) oxalyl chloride, DMAP (cat.), r.t.; b) 7-substituted indole **10**, Et_2_AlCl, CHCl_3_, 51-66%; viii. BCl_3_, CH_2_Cl_2_,0°C - r.t.; 80-85%; ix. 5% NaOH in methanol, reflux, 66-70%.

The synthesis strategy of hydroxyfuroic acids is summarized and presented in Fig. 2, as described below.

#### Furan diester 3

A 615 mL of benzene solution containing 170 mmol dimethyl diglycolate **1 **and 230 mmol indolylglyloxalate **2 **was added drop wise to a 720 mL of refluxing benzene suspension containing *t*-BuOK, prepared from dissolving potassium (4.5 molar eqs *vs 1*) in 765 mmol *t*-BuOH. After 2.5 h, the cooled mixture was acidified and then 500 mL of ethyl acetate was added while under stirring. The organic layer was separated, concentrated, added with 1550 mL of DMF, and then treated with MeI (572 mmol) and K_2_CO_3 _(770 mmol). After 21 h of stirring, residues in the mixture was filtered off and the filtrate was evaporated under reduced pressure, followed by partitioning with CH_2_Cl_2_/H_2_O. The separated organic layer was condensed and the residue was purified by chromatography on a silica gel to yield **3 **(76.5 mmol, 45%).

#### Furan diester 4

Fifty-seven mmol of **3 **and 110 mmol of DDQ was mixed in 813 mL of CH_2_Cl_2_/H_2_O (18:1, v/v) and stirred at room temperature for 21 h. The resulting mixture was quenched with 5% aqueous Na_2_CO_3_, followed by partitioning with CH_2_Cl_2_/H_2_O. The separated organic layer was condensed and the residue was purified by silica gel chromatography to yield **4 **(34.2 mmol, 60%).

#### Furan diester 5

A 460 mL 2-butanone solution containing 46 mmol **4 **was treated with TsCl (92 mmol) and K_2_CO_3 _(137 mmol). After 2 h refluxing, additional 46 mmol TsCl and 69 mmol K_2_CO_3 _were added and refluxed further overnight. The mixture was filtered and the filtrate was evaporated to leave a solidified residue. The residue was triturated over methanol to form a white powder suspension. The powder was isolated by filtration and dried to give **5 **(42.3 mmol, 92%). Alternatively, **5 **can be prepared by metal-catalyzed cross coupling of triflorosulfonated **6 **and an *N*-tosyl-3-iodoindole derived boric acid **7 **(1.5 molar eqs) in refluxing THF (20 mL/g) which had a 66% yield.

#### Furan diacid 8

A solution containing 25.4 mmol of **5 **in 280 mL methanol and 23 mL H_2_O was added with 177.7 mmol of lithium hydroxide at 25°C and stirred for 5 h. The mixture was acidified with 3 N HCl, followed by evaporation. The residue was extracted with ethyl acetate and washed with H_2_O. The separated organic layer was evaporated to give **8 **(25.2 mmol, 99%).

#### Furan halfester 9

Methyl chloroformate (22.0 mmol) predissolved in 30 mL dichloromethane was added to a 100 mL dichloromethane solution containing 22.0 mmol of **8 **and 23.1 mmol of triethylamine at 0°C. After 1 h stirring at 0°C, DMAP (2.6 mmol) was added and stirred at 0°C for 1 h and 18 h at room temperature. The resulting solution was then acidified, extracted with dichloromethane, and solvent evaporated. The residue was purified by silica gel chromatography to yield **9 **(13.2 mmol, 60%).

#### Furan bisiindoles 11

Oxalyl chloride (10 mL/g) was added to **9 **(12.2 mmol) and stirred for 20 minutes. The mixture was condensed under vacuum to give the acyl chloride of **9**. In a separated flask, a 60 mL dichloromethane solution containing 12.4 mmol of the 7'-substituted indoles **10 **was added with Et_2_AlCl (1 M in hexane, 30 mmol) at 0°C and stirred for 0.5 h before 50 mL dichloromethane solution containing the aforementioned acyl chloride **9 **was added. After 1 h stirring at 0°C and 14 h at room temperature, the reaction was quenched with 3 N HCl. The separated organic layer was condensed and the residue was purified by silica gel chromatography to yield **11 **(51-66% yield).

#### Furan bisindoles 12

A 50 mL dichloromethane solution containing 5.3 mmol **4 **was added with BCl_3 _(1 M in hexane, 35 mmol) at 0°C. The reaction mixture was stirred at 0°C for 0.5 h and at room temperature for 3 h, and then diluted with additional 200 mL dichloromethane. After another 0.5 h stirring, the mixture was quenched on ice water and the separated organic layer was condensed. The residue was purified by silica gel chromatography and yielded **12 (**80-85% yield).

#### *Bisindolylhydroxyfuroic acids *(D-410637-D-410639 and D-410641-D-410644)

A methanol solution (10-15 mL/g) containing 0.11 g of **12 **was added with 1.0 mL of 5% aqueous NaOH and refluxed for 0.5 h. The mixture was concentrated, acidified with 3 N HCl, and then extracted with ethyl acetate. The extract was washed with brine, dried, and solvent evaporated. The residue was purified by silica gel chromatography to give the titled compounds with 66-70% yield.

#### *Bisindolylhydroxythiophene carboxylic acid *(D-410640)

The compound was obtained with 9.5% overall yield by applying the same methods described in furoic acid derivative (D-410639) but using thiodiglycolic acid dimethyl ester and **2 **as the starting materials.

Physical properties of bisindolylhydroxyfuroic acid compounds are listed as following:

D-410637, a yellow powder, mp 140-142°C (ethyl acetate); ^1^H-NMR (500 MHz, ^6^d-DMSO) δ: 1.84-1.88 (m, 2H), 2.95 (t, J = 7.4 Hz, 2H), 3.51 (t, J = 6.5 Hz, 2H), 7.02-7.05 (m, 1H), 7.11-7.15 (m, 2H), 7.18-7.21 (m, 1H), 7.46 (d, J = 8.0 Hz, 1H), 7.50 (d, J = 8.0 Hz, 1H), 7.62-7.63 (m, 1H), 8.27 (d, J = 7.5 Hz, 1H), 8.66 (d, J = 2.2 Hz, 1H). Mass spectrometry (MS) (ESI, negative mode): 443.2 (M^+^-H); high resolution mass spectrometry (HREIMS): Calculated for C_25_H_20_N_2_O_6 _(M^+^) 444.4361, found 444.4358.

D-410638, a yellow powder, mp 145-147°C (ethyl acetate); ^1^H-NMR (500 MHz, ^6^d-acetone) δ: 1.63-1.65 (m, 2H), 1.84-1.86 (m, 2H), 3.01 (t, J = 7.5 Hz, 2H), 3.64 (t, J = 6.5 Hz, 2H), 7.07-7.08 (m, 1H), 7.15-7.17 (m, 2H), 7.23-7.25 (m, 1H), 7.68 (d, J = 8.0 Hz, 1H), 7.35-7.36 (m, 1H), 7.48 (d, J = 8.0 Hz, 1H), 7.79-80 (m, 1H), 8.35 (d, J = 8.0 Hz, 1H), 8.60 (br s, 1H), 8.90 (br s, 1H). MS (ESI, negative mode): 457.2 (M^+^-H); HREIMS: Calculated for C_26_H_22_N_2_O_6 _(M^+^) 458.1478, found 458.1459.

D-410639, a yellow powder, mp 158-160°C (ethyl acetate); ^1^H-NMR (500 MHz, ^6^d-acetone) δ: 1.53-1.55 (m, 2H), 1.61 (t, J = 7.2 Hz, 2H), 1.82 (t, J = 7.4 Hz, 2H), 3.01 (t, J = 7.8 Hz, 2H), 3.58 (t, J = 6.2 Hz, 2H), 7.10-7.11 (m, 1H), 7.18-7.20 (m, 2H), 7.26-7.28 (m, 1H), 7.51 (d, J = 8.0 Hz, 1H), 7.69 (d, J = 8.0 Hz, 1H), 7.79 (d, J = 2.5 Hz, 1H), 8.38 (d, J = 7.9 Hz, 1H), 8.84 (d, J = 3.2 Hz, 1H), 10.7 (br s, 1H), 11.4 (br s, 1H). MS (ESI): 473.0 (M^+^+H); HREIMS: Calculated for C_27_H_24_N_2_O_6 _(M^+^) 472.1634, found 472.1639.

D-410640, a yellow powder, mp 144-146°C (ethyl acetate); ^1^H-NMR (^6^d-DMSO) δ: 1.39-1.40 (m, 2H), 1.47-1.49 (m, 2H), 1.66-1.69 (m, 2H), 2.91 (t, J = 7.6 Hz, 2H), 3.39 (t, J = 7.1 Hz, 2H), 6.95-6.98 (m, 1H), 7.06-7.10 (m, 2H), 7.15-7.18 (m, 1H), 7.39-7.43 (m, 2H), 7.52 (s, 1H), 8.13 (d, J = 7.9 Hz, 1H), 8.41 (s, 1H), 11.2 (br s, 1H), 12.4 (br s, 1H), 13.2 (br s, 1H). MS (ESI): 489.0 (M^+^+H); HREIMS: Calculated for C_27_H_24_N_2_O_5_S (M^+^) 488.1406, found 488.1421.

D-410641, an amorphous oil; ^1^H-NMR (^4^d-MeOH) δ: 0.90 (m, 3H), 1.39 (br s, 4H), 1.73 (br s, 2H), 2.90 (br s, 2H), 7.04 (m, 2H), 7.11 (m, 1H), 7.17 (m, 1H), 7.39 (d, J = 8.1 1H), 7.59 (d, J = 8.7, 2H), 8.28 (d, J = 7.8 Hz, 1H), 8.86 (br s, 1H). MS (ESI): 457.0 (M^+^+H); HREIMS: Calculated for C_27_H_24_N_2_O_5 _(M^+^) 456.1685, found 456.1693.

D-410642, a yellow powder, mp 141-143°C (ethyl acetate); ^1^H-NMR (500 MHz, ^6^d-acetone) δ: 1.50-1.53 (m, 2H), 1.74-1.76 (m, 1H), 1.85-1.89 (m, 2H), 3.01 (t, J = 7.9 Hz, 2H), 3.63-3.67 (m, 4H), 7.09-7.12 (m, 1H), 7.17-7.21 (m, 2H), 7.25-7.28 (m, 1H), 7.51 (d, J = 8.1 Hz, 1H), 7.68 (d, J = 8.0 Hz, 1H), 7.79 (d, J = 2.6 Hz, 1H), 8.38 (d, J = 7.8 Hz, 1H), 8.84 (d, J = 3.1 Hz, 1H), 10.68 (br s, 1H),11.52 (br s, 1H). MS (ESI): 503.0 (M^+^+H); HREIMS: Calculated for C_28_H_26_N_2_O_7 _(M^+^) 502.1740, found 502.1746.

D-410643, an amorphous oil; ^1^H-NMR (500 MHz, ^6^d-acetone) δ: 1.74 (d, J = 7.6 Hz, 2H), 1.92 (d, J = 2.1 Hz, 2H), 2.38 (t, J = 7.5 Hz, 2H), 3.02 (t, J = 7.5 Hz, 2H), 7.08-7.11 (m, 1H), 7.17-7.21 (m, 2H), 7.25-7.27 (m, 1H), 7.50 (d, J = 8.1 Hz, 1H), 7.67 (d, J = 7.8 Hz, 1H), 7.77 (d, J = 2.4 Hz, 1H), 8.37 (d, J = 7.8 Hz, 1H), 8.83 (d, J = 3.0 Hz, 1H), 10.66 (br s, 1H),11.44 (br s, 1H). MS (ESI): 487.0 (M^+^+H); HREIMS: Calculated for C_27_H_22_N_2_O_7 _(M^+^) 486.1427, found 486.1432.

D-410644, an amorphous oil; ^1^H-NMR (^6^d-acetone) δ: 3.25 (t, J = 7.0 Hz, 2H), 3.6 (t, J = 7.0 Hz, 2H), 7.09 (m, 1H), 7.17 (m, 1H), 7.26 (m, 2H), 7.50 (d, J = 8.1 Hz, 1H), 7.67 (d, J = 8.0 Hz, 1H), 7.77 (d, J = 2.6 Hz, 1H), 8.39 (d, J = 7.6 Hz, 1H), 8.85 (d, J = 3.1 Hz, 1H), 10.7 (br s, 1H), 11.5 (br s, 1H). MS (ESI): 459.0 (M^+^+H). HREIMS: Calculated for C_25_H_18_N_2_O_7 _(M^+^) 458.1114, found 458.1109.

### Insulin Receptor Activation Assay

The insulin receptor activation assay is based on the assay described by Zhang *et al. *[19-21] with minor modifications. Briefly, approximately 100,000 CHO cells overexpressing human recombinant insulin receptor were suspended in 100 μl of Ham's F-12 medium containing 10% fetal bovine serum (FBS) and seeded into each well of a 96-well plate. Cells were incubated at 37°C with 5% CO_2 _aeration overnight. CHO cells were rinsed with phosphate buffered saline (PBS) 3 times and starved in serum free Ham's F-12 medium for 2 hours before being stimulated with desired concentration of insulin or test compound for 20 minutes. Test compounds were predissolved in DMSO as 100× stocks and serially diluted when necessary. Stimulated CHO cells were then rinsed 3 times with PBS and lysed in 60 μl of lysis buffer. Fifty μl of the lysate was transferred to a well on a Flexible Assay Plate that was precoated with Ab-3 antibody recognizing the β-subunit of insulin receptor. The well was then rinsed 3 times with Tris buffered saline (TBS) containing 0.1% Tween20 and thrown dried by hand before 10 μl of tyrosine kinase reaction mixture (50 mM HEPES, pH7.4, 5 mM MgCl_2_, 5 mM MnCl_2_, 1 mg/mL polyGlu/Tyr and 250,000 cpm γ-P^33 ^ATP/well) was added. The reaction was carried out at 25°C for 40 minutes and terminated by adding 50 μl ice cold 100 mM phosphoric acid. Fifty μl of the stopped reaction mixture was then transferred to an inch square P81 paper. The P81 paper was air dried and then rinsed in Millipore water 5 times to remove leftover radioactive ATP and product ADP. Five mL of scintillation cocktail was added to the paper before the count was read in a liquid scintillation counter.

### IGF-1 Receptor Tyrosine Kinase Activity Assay

The reaction was carried out at pH7.0 in the presence of 50 mM HEPES, 5 mM MgCl_2_, 0.01 μg/μl polyGlu/Tyr substrate, 5 mM DTT, 250,000 cpm radioactive ATP, and 0.4 ng/μl IGF-1R tyrosine kinase with a final volume of 100 μl at 37°C for 10 minutes. The IGF-1 receptor tyrosine kinase reaction was carried out with subsaturating concentrations of substrates for better sensitivity to activator or inhibitor influence. Test compound D-410639 was predissolved in DMSO as 20× stock. The reaction was stopped by the addition of 100 μl of 25 mM EDTA (pH8), and 85 μl of the stopped reaction mix was blotted on an inch square P81 paper. P81 paper was air dried before rinsed 5 times in Millipore water to remove non-protein phosphorus. Five mL scintillation cocktail was added to the P81 paper before the counts were read with a liquid scintillation counter.

### EGF Receptor (ErbB1) Tyrosine Kinase Activity Assay

The reaction condition of EGF receptor (ErbB1) was similar to that of IGF-1 receptor reaction except 0.04 μg/μl polyGlu/Tyr substrate and 0.3 ng/μl EGF receptor (ErbB1) were used, and the reaction time was 15 minutes. All other conditions or processes were the same. The EGF receptor tyrosine kinase reaction was carried out with subsaturating concentrations of substrates for better sensitivity to activator or inhibitor influence.

### FGF Receptor 3 Tyrosine Kinase Activity Assay

The reaction condition of FGF receptor 3 was similar to that of EGF receptor except 0.1 μg/μl polyGlu/Tyr substrate and 0.05 ng/μl FGF-receptor 3 were used instead. All other conditions or processes were the same. The FGF receptor 3 tyrosine kinase reaction was carried out with subsaturating concentrations of substrates for better sensitivity to activator or inhibitor influence.

### Cytotoxicity Assay

Approximately 5,000 CHO cells suspended in 100 μl D-MEM containing 10% FBS were seeded in each well of a 96-well plate overnight before the cytotoxicity assay. Test compounds were predissolved as 10× solution in 10% DMSO, 10% ethanol, 80% PBS and 10 μl of this 10× test compound solution were added to each well. After 24 hours incubation, medium containing test compound was removed by aspiration and 3 rinses of PBS. One hundred μl of serum medium was replenished before 20 μl methylthiazolyldiphenyl-tetrazolium bromide (MTT, 2.5 mg/mL) and 1 μl phenazine methosulfate (PMS, 50 μM) was added to each well before a 3-hour further incubation at 37°C. Optical density at 450 nm was measured with an ELISA reader. LC_50 _is defined as the concentration of compound that causes 50% drop of cell viability in this test.

### Determination of EC_50 _or IC_50_

The 50% effective or inhibition concentrations were determined with the standard curve analysis of SigmaPlot 8.02. The nonlinear regression equation is y = min+(max-min)/(1+(x/EC_50_)^Hill Slope^) where y is the observed responses; x is the dose concentrations; max and min are approximated by the program automatically during the calculation.

## Results

### Insulin receptor tyrosine kinase activation

To validate the insulin receptor activation assay, dose response curve of bovine insulin was determined. The EC_50 _of bovine insulin is approximately 5.1 nM, which is reasonable comparing to 1 nM reported by Liu *et al. *[21]. In subsequent experiments, we routinely included 1, 10, and 100 nM insulin as standard reference. Since 10 nM insulin showed more consistent potency, we chose 10 nM insulin as the standard concentration serving as a reference point for all experiments. For simplicity, the efficacies of hydroxyfuroic acid compounds were expressed as percentage of activities comparing to the response provided by 10 nM insulin stimulation in parallel. To distinguish human insulin receptor kinase from other tyrosine kinases in CHO cells, Ab-3, a human insulin receptor β-subunit specific monoclonal antibody, was used to absorb insulin receptor from the CHO cell lysate, so the radioactivity catalyzed by captured tyrosine kinase is attributable to insulin receptor tyrosine kinase activity.

### Non-quinone hydroxyfuroic acid as insulin receptor activator

Various hydroxyalkyl side chains were derived to test the best structure on the 7-indolyl position. Hydroxyl or carboxyl derivatives of 3-carbon or longer side chain showed sustained insulin receptor activation potential (Table 1). A side chain of 5-carbon alcohol showed better efficacy (D-410639, 72-89%) over 4- or 3- carbon alcohol derivatives (D-410638, 31.3%; D-410637, 18.9%, respectively). The hydroxyl group is important because its non-hydroxyl counterpart (D-410641) achieved only 48.6% efficacy. One additional hydroxymethyl side chain (D-410642) does not help the efficacy, since it remains low at 40.4%; nor oxidation of the hydroxyl group to a carboxyl group, because it offers no substantial improvement to the efficacies (D-410643, 48.9%; D-410644, 21.9%, respectively). Therefore, a 5-carbon hydroxypentyl side chain on the 7-indolyl position offers the best insulin receptor activation efficacy thus far, and consistently we have observed highest receptor responses associated with D-410639 stimulation when compared to other compounds in multiple occasions (data not shown).

**Table 1:** Relative insulin receptor activation potentials of hydroxyfuroic acid compounds with various prenylindole moiety.

Compound	Side Chain	Study 1^a^	% activation^b^	Study 2^a^	% activation^b^
D-410637	(CH_2_)_2_CH_2_OH	18.9			
D-410638	(CH_2_)_3_CH_2_OH	31.3			
D-410639	(CH_2_)_4_CH_2_OH	5590	89.4	1181	72.3
D-410641	(CH_2_)_4_CH_3_			792.7	48.6
D-410642	(CH_2_)_3_CH(CH_2_OH)_2_			659.3	40.4
D-410643	(CH_2_)_4_COOH			799	48.9
D-410644	(CH_2_)_2_COOH			357.3	21.9

Insulin - 10 nM		6250		1632.5	

^a ^Data in these columns are cpm, representing phosphoryl incorporation by tyrosine kinase activities. Study 1 and 2 are separate experiments each with a 10 nM insulin and a 100 μM D-410639 control for internal reference. The test concentration for hydroxyfuroic acid compounds was 100 μM. See Materials and Methods for detail.

^b ^Percentage of tyrosine kinase activities as compared to that offered by 10 nM insulin stimulation, presented at the bottom row.

**Figure 3:**
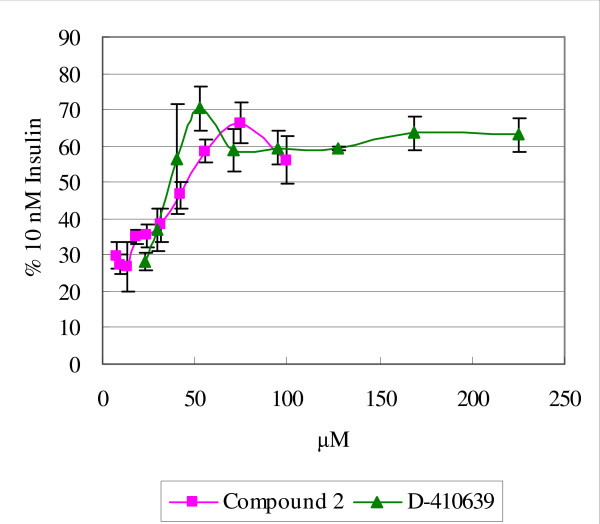
**Insulin receptor tyrosine kinase activation curves by compound D-410639 and compound 2**. The activation assays were determined side by side, and both compounds plateau out at about 60% level achieved by 10 nM insulin with EC_50 _being 32.9 μM for D-410639 and 38.9 μM for compound 2. Error bars indicate standard deviations.

In a separate experiment, the potency of D-410639 was compared to that of compound 2, as shown in Fig. 3. In this assay, the maximal activity of either compound reached 66-70% of receptor kinase activation provided by 10 nM insulin and the EC_50 _are 33 and 39 μM for D-410639 and compound 2, respectively. The EC_50 _for compound 2 is about 2-order different from 0.3 μM reported previously by Merck's group [20,21]. The exact reason for this discrepancy is not clear, as our compound 2 was also obtained from Merck, however, a logical explanation is that different laboratory practice resulted in different outcomes but under identical conditions, D-410639 is as potent as compound 2.

**Figure 4:**
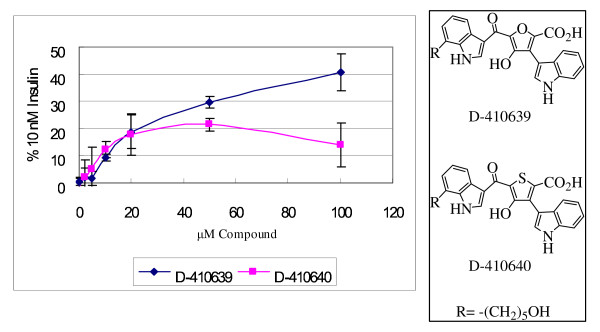
**Dose response curves of compound D-410639 and D-410640**. Receptor tyrosine kinase activation was normalized against 10 nM insulin activation. Each curve is an average of 3 determinations and the error bars indicate standard deviations.

D-410640 is a thiophene equivalent of D-410639, with a higher yield advantage during the synthesis process, so its efficacy on insulin receptor activation was examined. Unfortunately, the efficacy of D-410640 levels off at 50 μM and tapers off by 100 μM, suggesting a negative effect associated with the thiophene moiety at mid to high μM range. As a result, thiophene derivatives were not pursued further (Fig. 4).

### Cytotoxicity and growth factor receptor selectivity of D-410639

**Figure 5:**
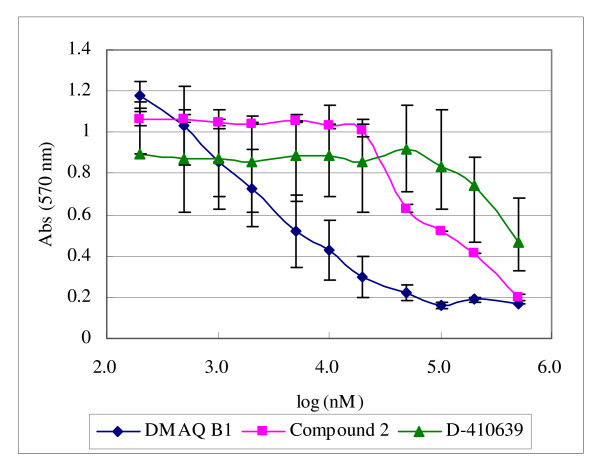
**Cytotoxicity test of DMAQ-B1, compound 2 and D-410639 on CHO cells**. CHO cells were treated with various concentrations of test compounds and their viability was measured with the use of MTT. LC_50 _for DMAQ-B1, compound 2, and D-410639 are 2.3 μM, 66.5 μM, and 295.9 μM, respectively. Error bars indicate standard error of the mean (SEM). See Materials and Methods for detail.

Because D-410639 is a non-quinone derivative, its cytotoxicity against quinone derivatives, *i.e.*, DMAQ B1 and compound 2, on Chinese hamster ovary (CHO) tissue cell culture were examined. DMAQ B1 showed the strongest cytotoxic effect (LC_50 _= 2.3 μM), while compound 2 improved the cytotoxicity to 66.5 μM. As one may expect, D-410639 further alleviated the cytotoxicity to approximately 296 μM, attributable to the replacement of the dihydroxyquinone moiety with a hydroxyfuroic acid moiety (Fig. 5).

**Figure 6:**
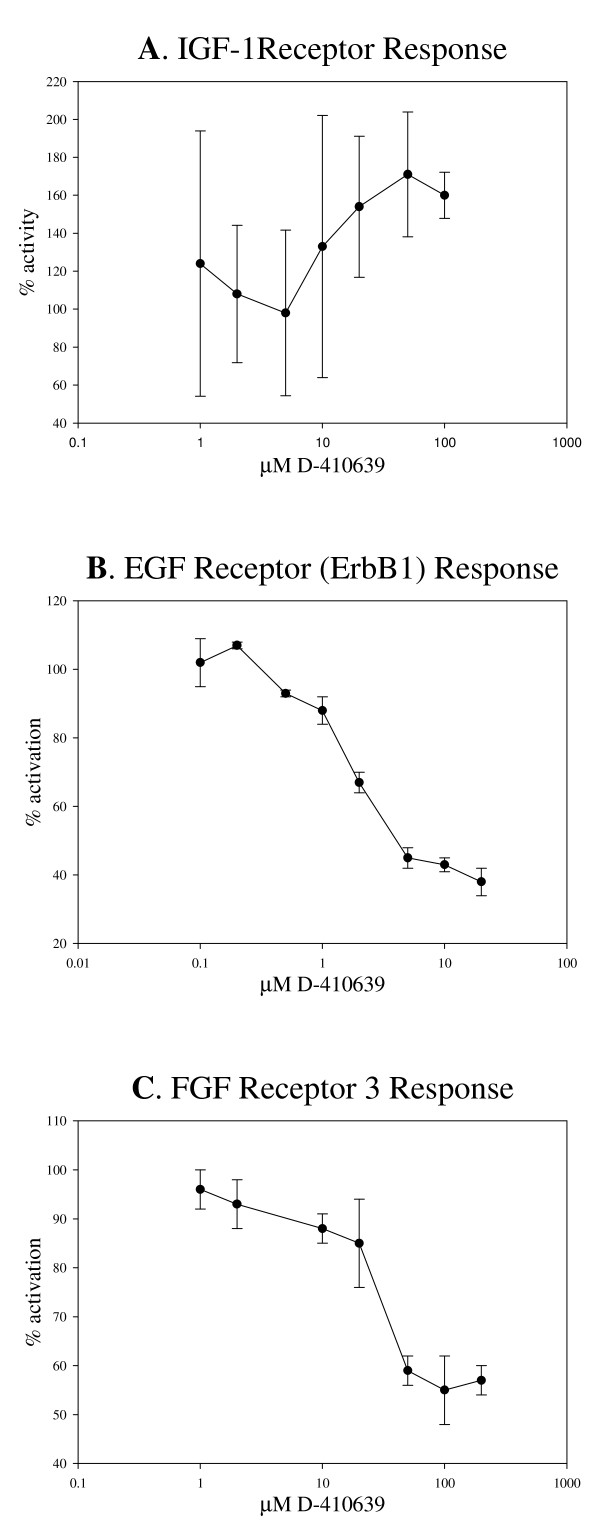
**Dose effect of compound D-410639 on the tyrosine kinase activities of IGF-1 receptor (panel A), EGF receptor (ErbB1, panel B), and FGF receptor 3 (panel C)**. Receptor tyrosine kinase activation was compared to the control which received a blank placebo, and the base line control was considered as 100% activity. Error bars indicate standard deviations. Projected EC_50 _for IGF-1 receptor was 16.9 μM, but the EC_50 _is not statistically significant. The IC_50 _for EGF receptor (ErbB1) and FGF receptor 3 are 1.8 μM and 26.5 μM, respectively. See Materials and Methods for detail.

Asterriquinone are known for its potential to activate insulin-like growth factor-1 (IGF-1) and epidermal growth factor (EGF) receptors at mid to high μM range [19,22]; therefore, it is important to characterize D-410639's activation potential at these growth factor receptors. The IGF-1 receptor activity was increased by about 60-70% from the base line with mid μM of D-410639 (Fig. 6A) and the non-linear EC_50 _fitting curve program suggested EC_50 _to be 17 μM, but the statistical t-test of the EC_50 _itself is not significant (p = 0.63) due to large variation.

Unlike the minor activation potential of DMAQ-B1 on EGF receptor (ErbB1) as reported by Zhang *et al. *[19], D-410639 demonstrated a substantial inhibitory property on EGF-R (ErbB1) tyrosine kinase activity, with the IC_50 _being estimated to be 1.8 μM (Fig. 6B). Interestingly, D-410639 is also an effective inhibitor on fibroblast growth factor (FGF) receptor 3 tyrosine kinase with the IC_50 _projected at 26.5 μM (Fig. 6C).

## Discussion

This study presents a new class of insulin receptor activator, compounds with a hydroxyfuroic acid scaffold, which, along with DMAQ B1 and Merck's compound 2, can activate insulin receptor in the absence of insulin. In contrast to the report by Wood *et al. *[27], we found the dihydroxyquinone moiety is replaceable by a hydroxyfuroic acid moiety without losing much of the insulin receptor activation capability (Fig. 3). At the meantime, without a dihydroxyquinone moiety, the cytotoxicity effect of insulin receptor activator on CHO cells was reduced by 128 fold (Fig. 5) as compared to DMAQ B1.

Perhaps the most intriguing part of these new insulin receptor activators is that dihydroxyquinone derivatives can tolerate the replacement of isoprenylindole by a phenyl group so well that compound 2 of Merck has an even better efficacy than original DMAQ B1 [20,21]. However, the same does not apply to hydroxyfuroic acid compounds; a phenyl substitution for the isoprenylindole on a hydroxyfuroic acid compound renders these compounds inactive completely. That is, DMAQ B1 may be transformed to a hydroxyfuroic acid derivative or simplified to compound 2 (phenylindolylhydroxyquinone) and retains its insulin receptor activation potential, but these two features cannot be combined together (Fig 2). The reason for this incompatibility may be too difficult to be demonstrated by the medicinal chemistry approach; perhaps X-ray crystallography of these compounds with an insulin receptor may have a better chance of answering this question. Despite insulin receptor tyrosine kinase domain structure has been solved long ago [28] and the activation mechanism is well studied [29], the exact binding pocket for DMAQ B1 on insulin receptor remains unknown. Though this site has been postulated [30], how does the binding of an insulin receptor activator compound trigger receptor tyrosine kinase activation remains to be illustrated.

The prenylindole moiety is important for bioactivities of asterriquinone and compounds with a hydroxyfuroic acid scaffold (Table 1), because: i) diisoprenylindolylasterriquinone (L-767827 in ref. [19]) showed no insulin receptor tyrosine kinase activation potential; ii) in a series of test compounds (D-410637, D-410638 and D-410639), the compound's efficacy is dictated by the length of the hydroxyalkyl chain, which is an oxidized derivative of the prenyl chain. Presumably, this alkyl side chain may have a corresponding binding pocket on the insulin receptor, and the pocket is probably hydrophobic in nature because pentanol (D-410639) or pentyl (D-410641) side chains are both well accommodated while shorter butanol (D-410638) or propanol (D-410637) side chains are well tolerated but with lower efficacies. At the distal end of this side chain pocket, insulin receptor prefers one hydroxyl group because the activity improves with the addition of a hydroxyl group on the pentyl chain, but the addition of a second hydroxymethyl group (D-410642) or oxidation of the terminal alcohol group to a carboxyl group (D-410643) offers no improvement. Therefore, a hydrophilic group and potentially a hydrogen bond donor or acceptor is likely preferred at the distal end of this pocket. However, compound 2 of Merck is a noticeable exception which does not have a prenyl or an alkyl chain on its methylindole moiety. How does compound 2 activate insulin receptor tyrosine kinase without a 7-prenyl or 7-alkyl chain remains to be illustrated.

The exact nature or importance of the isoprenyl chain on DMAQ B1 is not known, since we did not have any compound for direct comparison. But the isoprenylindole moiety can be simplified to an indole and still retains significant activity (D-410639 and all other compounds listed in Table 1), suggesting it is not essential for insulin receptor activation with a hydroxyfuroic acid as the scaffold. If this assumption is false, all those hydroxyfuroic acid compounds in Table 1 would have shown no activity essentially. Therefore, it is suggested that isoprenyl group is not likely to play a significant role as other features at activating insulinreceptor.

Another interesting aspect of D-410639 is its inhibitory potential at epidermal growth factor receptor. Studies have shown that EGF signal plays a major role at the development of vascular dysfunction in diabetic animals [31,32], and thus inhibition of EGF receptor in diabetic subjects may be useful at alleviating vascular complication associated with the hyperglycemic condition [33]. Therefore, a dual function compound like D-410639 with dual properties, activator for insulin receptor and antagonist for EGF receptor, is rather interesting and deserves further study.

## Abbreviations

CHO: Chinese hamster ovary; DDQ: 2,3-dichloro-5,6-dicyano-*p*-benzoquinone; DMAP: 4-dimethylaminopyridine; DMAQ B1: demethylasterriquinone B1; DMF: *N,N*-dimethylformamide; D-MEM: Dulbecco's modified Eagle's medium; DMSO: dimethylsulfoxide; EGF: epidermal growth factor; FBS: fetal bovine serum; FGF: fibroblast growth factor; HRMS: high resolution mass spectrometry; IGF-1: insulin-like growth factor-1; MTT: methylthiazolyldiphenyl-tetrazolium bromide; MS: mass spectrometry; PBS: phosphate buffered saline; PMS: phenazine methosulfate; TBS: tris buffered saline.

## Competing interests

The authors declare that they have no competing interests.

## Authors' contributions

HJT is responsible for biological assays; SYC is responsible for chemical syntheses.
